# PreMedKB: an integrated precision medicine knowledgebase for interpreting relationships between diseases, genes, variants and drugs

**DOI:** 10.1093/nar/gky1042

**Published:** 2018-11-08

**Authors:** Ying Yu, Yunjin Wang, Zhaojie Xia, Xiangyu Zhang, Kailiang Jin, Jingcheng Yang, Luyao Ren, Zheng Zhou, Dong Yu, Tao Qing, Chengdong Zhang, Li Jin, Yuanting Zheng, Li Guo, Leming Shi

**Affiliations:** 1 State Key Laboratory of Genetic Engineering, School of Life Sciences and Shanghai Cancer Hospital/Cancer Institute, Fudan University, Shanghai 200438, China; 2 State Key Laboratory of Multiphase Complex Systems, Institute of Process Engineering, Chinese Academy of Sciences, Beijing 100190, China; 3 JD.com Co., Ltd., Beijing 100080, China; 4 State Key Laboratory of Genetic Engineering, Collaborative Innovation Center for Genetics and Development, School of Life Sciences, Fudan University, Shanghai 200438, China; 5 Human Phenome Institute, Fudan University, Shanghai 201203, China; 6 Fudan-Gospel Joint Research Center for Precision Medicine, Fudan University, Shanghai 200438, China; 7 School of Chemical Engineering, University of Chinese Academy of Sciences, Beijing 100049, China

## Abstract

One important aspect of precision medicine aims to deliver the right medicine to the right patient at the right dose at the right time based on the unique ‘omics’ features of each individual patient, thus maximizing drug efficacy and minimizing adverse drug reactions. However, fragmentation and heterogeneity of available data makes it challenging to readily obtain first-hand information regarding some particular diseases, drugs, genes and variants of interest. Therefore, we developed the Precision Medicine Knowledgebase (PreMedKB) by seamlessly integrating the four fundamental components of precision medicine: diseases, genes, variants and drugs. PreMedKB allows for search of comprehensive information within each of the four components, the relationships between any two or more components, and importantly, the interpretation of the clinical meanings of a patient's genetic variants. PreMedKB is an efficient and user-friendly tool to assist researchers, clinicians or patients in interpreting a patient's genetic profile in terms of discovering potential pathogenic variants, recommending therapeutic regimens, designing panels for genetic testing kits, and matching patients for clinical trials. PreMedKB is freely accessible and available at http://www.fudan-pgx.org/premedkb/index.html#/home.

## INTRODUCTION

Precision medicine refers to the medical model that tailors an individual patient's pan-omic data, lifestyle and environment to analyze the disease pathogenicity at the molecular level and then to utilize targeted treatments (possibly in combination) to address that individual patient's disease process. One important aspect of precision medicine aims to deliver the right medicine to the right patient at the right dose at the right time based on the unique ‘omics’ features of each individual patient, thus maximizing drug efficacy and minimizing adverse drug reactions (1–4). Ever since the concept of precision medicine emerges, it has shown the potential of profoundly improving the practice of medicine, as well as promoting the pace of drug development and gaining insight into genetic diseases (5–7). Being data-driven in nature, precision medicine deeply relies on the robustness of sequencing technology, data analysis methods and knowledge to interpret the clinical meanings of genomic variants. It has been well recognized that clinical interpretation of the genomic variants underlying a patients’ disease is the bottleneck of the workflow of precision medicine (8).

Great efforts have been made by researchers to develop a growing number of genomic tools and databases to facilitate the interpretation of genomic variants, such as My Cancer Genome (http://www.mycancergenome.org), ANNOVAR (9), Clinical Interpretation of Variants in Cancer (CIViC) (https://civicdb.org/) (10), The Human Gene Mutation Database (HGMD) (http://www.hgmd.cf.ac.uk/ac/index.php) (11), the Human Genome Variation Archive (HGVA) (12), the Pharmacogenomics Knowledgebase (PharmGKB) (https://www.pharmgkb.org/) (13) and Therapeutic Target Database (TTD) (http://bidd.nus.edu.sg/group/cjttd/) (14). These databases gather knowledge on approved or potential therapies in cancer or other diseases, disease pathogenicity, pharmacogenomics and drug development that requires to maximize the power of precision medicine. However, these independent resources are scattered in different websites and usually cannot interoperate with each other, making it challenging for clinicians, geneticists, biologists and patients to obtain the first-hand information and maximize the added-value of the diverse data resources (15). Furthermore, facing a large number of variants derived from the sequencing analysis results of different patients, there is an urgent need for an infrastructure to perform simple, quick, and routine annotations of multi-dimensional data.

Therefore, we developed the Precision Medicine Knowledgebase (PreMedKB) by seamlessly integrating the well-established data sources incorporating the four fundamental components of precision medicine: diseases, genes, variants and drugs, thus allowing for search of comprehensive information within each for the four components, the relationships between any two or more components, and importantly, the interpretation of the clinical meanings of a patient's genetic variants. The reliability of the PreMedKB system has been extensively tested and confirmed with the interpretation of the genetic profiles of thousands of patients from our ongoing research projects. And more recently, the performance of the PreMedKB system has been compared favorably with that of the OncoKB used for interpreting the MSK-IMPACT data set of genetic profiles of ∼10 000 cancer patients (16,17). PreMedKB is freely accessible and available at http://www.fudan-pgx.org/premedkb/index.html#/home. We recommend using modern browsers, for example, Chrome, Firefox, Safari and IE 10 or higher version, to access PreMedKB for better performance.

## MATERIALS AND METHODS

### PreMedKB architecture

**Figure 1.**
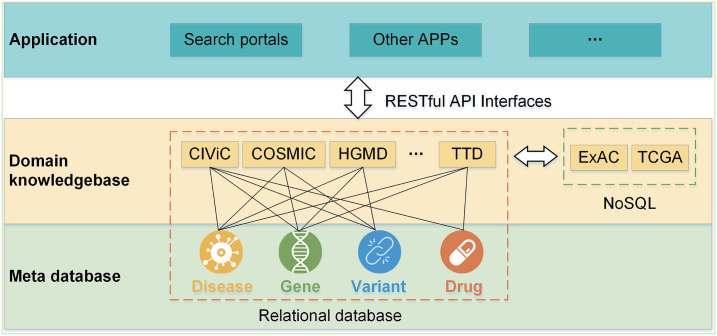
PreMedKB architecture with three layers including meta databases, domain knowledgebases, and application modules. The meta database layer consists of databases on diseases, genes, variants and drugs with their respective metadata such as names and synonymies. The domain knowledgebases consist of the relationships between two or more of the four components. Through RESTful APIs, application layer, consisting of user-friendly applications, can be connected with the meta database layer and the domain knowledgebase layer.

The architecture of PreMedKB is composed of three layers, i.e. the meta database layer, the domain knowledgebase layer and the application layer (Figure 1).

The meta database layer consists of databases on diseases, genes, variants, and drugs with their respective metadata such as names, synonymies, functions, and so on. The domain knowledgebases are those data sources containing the knowledge in interpreting the clinical meanings of diseases, genes, variants, or drugs. Their entries usually consist of the relationships between two or more of the four components. In one word, a meta database is used to describe the characteristics of each component, whereas the domain knowledgebase is to describe their relationships.

Application layer consists of user-friendly applications to visit PreMedKB, for example search portals. Through REpresentational State Transfer (RESTful) Application Program Interfaces (APIs), application layer can be connected with meta database layer and domain knowledgebase layer. Other applications can be flexibly docked with PreMedKB through APIs. Currently, APIs are designed for internal use, but not ready for the general public so far.

### Data sources

In order to present the most comprehensive landscape of our knowledge of precision medicine, PreMedKB integrated diverse and reliable data from expert-curated databases including the following resources, as shown in Table 1.

**Table 1.** Data sources and summary of integrated data

Meta database	Data sources
Disease meta data	Disease Ontology, ICD-10, MalaCards, OMIM, Orphanet
Gene meta data	HGNC, NCBI.Gene, OMIM, UniProtKB, GTEx, TCGA
Variant meta data	CIViC, COSMIC, ClinVar, dbSNP, HGMD, ExAC
Drug meta data	Drugs@FDA, DrugBank, PubChem, STITCH, ClassyFire, DailyMed, TTD
	
Domain knowledgebase	Data sources
Semantic relation data source	CIViC, COSMIC, ClinVar, FDA Pharmacogenomic Biomarkers, HGMD, My Cancer Genome, NCCN, PharmGKB, TTD
Clinical trial support	ClinicalTrials
Literature support	MEDLINE, PubTator
Terminology	UMLS

Some of the abbreviations, their full names of above databases and URLs:

CIViC, Clinical Interpretations of Variants in Cancer, https://civicdb.org/;

ClassyFire, chemical classification, http://classyfire.wishartlab.com/;

ClinicalTrials, https://clinicaltrials.gov/;

ClinVar, Clinical Variation database, https://www.ncbi.nlm.nih.gov/clinvar/;

COSMIC, Catalogue Of Somatic Mutations In Cancer, https://cancer.sanger.ac.uk/cosmic;

dbSNP, single nucleotide polymorphism database, https://www.ncbi.nlm.nih.gov/snp;

DailyMed, https://www.dailymed.nlm.nih.gov/;

Disease Ontology, http://disease-ontology.org/;

DrugBank, https://www.drugbank.ca/;

Drugs@FDA, https://www.accessdata.fda.gov/scripts/cder/daf/;

ExAC, Exome Aggregation Consortium, http://exac.broadinstitute.org/;

FDA Pharmacogenomic Biomarkers, https://www.fda.gov/Drugs/ScienceResearch/ucm572698.htm;

GTEx, Genotype-Tissue Expression, https://gtexportal.org/;

HGMD, Human Gene Mutation Database, http://www.hgmd.cf.ac.uk/ac/index.php;

HGNC, HUGO Gene Nomenclature Committee, https://www.genenames.org/;

ICD-10, the 10th revision of the International Classification of Diseases and Related Health Problems (ICD), http://apps.who.int/classifications/icd10/browse/2016/en;

MalaCards, database of human maladies, https://www.malacards.org/;

MEDLINE, https://www.nlm.nih.gov;

My Cancer Genome, http://www.mycancergenome.org;

NCBI.Gene, https://www.ncbi.nlm.nih.gov/gene/;

NCCN, the National Comprehensive Cancer Network, https://www.nccn.org/;

OMIM, Online Mendelian Inheritance in Man, https://omim.org/;

Orphanet, rare diseases database, https://www.orpha.net/;

PharmGKB, the Pharmacogenomics Knowledgebase, https://www.pharmgkb.org/;

PubChem, https://pubchem.ncbi.nlm.nih.gov/;

PubTator, a web-based system for assisting biocuration, https://www.ncbi.nlm.nih.gov/CBBresearch/Lu/Demo/PubTator/;

STITCH, search tool for interactions of chemicals, http://stitch.embl.de/;

TCGA, The Cancer Genome Atlas, https://cancergenome.nih.gov/;

TTD, Therapeutic Target Database, http://bidd.nus.edu.sg/group/cjttd/;

UMLS, Unified Medical Language System, http://www.medical-language-international.com/;

UniProtKB, The Universal Protein Resource (UniProt) knowledgebase; https://www.uniprot.org/

#### Diseases

Information on diseases was mainly from Disease Ontology (http://disease-ontology.org/), the International Classification of Diseases, 10th Revision (ICD-10) (http://apps.who.int/classifications/icd10/browse/2016/en), MalaCards (https://www.malacards.org/) (18), Online Mendelian Inheritance in Man (OMIM) (https://omim.org/) (19), rare diseases database Orphanet (https://www.orpha.net/) (20) and Unified Medical Language System (UMLS) (http://www.medical-language-international.com/) (21).

#### Genes

Information on genes was mainly from HUGO Gene Nomenclature Committee (HGNC) (https://www.genenames.org/) (22), the gene information at NCBI (https://www.ncbi.nlm.nih.gov/gene/) (23), OMIM (19) and the universal protein knowledgebase (UniProtKB) (https://www.uniprot.org/) (24). Genomic landscapes and expression profile annotations were extracted from The Cancer Genome Atlas (TCGA) (https://cancergenome.nih.gov/) (25) and the Genotype-Tissue Expression (GTEx) project (https://gtexportal.org/) (26), which are data resources of multiple cancer genomes in multiple cancer tissues and normal samples in humans, respectively.

#### Variants

Information on variants was mainly from CIViC (10), Catalogue Of Somatic Mutations In Cancer (COSMIC) (https://cancer.sanger.ac.uk/cosmic) (27), clinical variation database ClinVar (https://www.ncbi.nlm.nih.gov/clinvar/) (28), dbSNP (https://www.ncbi.nlm.nih.gov/snp) (29) and HGMD (11). Variant allele frequencies computed by the Exome Aggregation Consortium (ExAC) (http://exac.broadinstitute.org/) (30) was used for population frequency annotation. In addition, we used ANNOVAR (9) and Ensembl Variant Effect Predictor (31) to annotate SNVs and INDELs with functional effects of the variants, allele frequencies in different populations and genome features such as CytoBand.

#### Drugs

Drug names and synonymies were originally integrated from databases on drugs such as Drugs@FDA (https://www.fda.gov/Drugs/InformationOnDrugs/ucm135821.htm), DrugBank (https://www.drugbank.ca/) (32), chemical database PubChem (https://pubchem.ncbi.nlm.nih.gov/) (33), Search Tool for Interactions of Chemicals (STITCH) (http://stitch.embl.de/) (34) and UMLS (21). Information about a drug, including structure, pharmacology, pharmacogenomics, clinical phase and product information, was integrated from DrugBank (32), marketed drug database DailyMed (https://www.dailymed.nlm.nih.gov/) (35), chemical classification database ClassyFire (http://classyfire.wishartlab.com/) (36), Drugs@FDA and TTD (14).

#### Relationships

In addition to the four types of meta databases described above, a series of databases were then used to annotate the relationships between any two or more components (diseases, genes, variants, and drugs). These databases include CIViC (10), COSMIC (27), ClinVar (28), FDA Pharmacogenomic Biomarkers (https://www.fda.gov/Drugs/ScienceResearch/ucm572698.htm), HGMD (11), My Cancer Genome, the National Comprehensive Cancer Network (NCCN) (https://www.nccn.org/), PharmGKB (13) and TTD (14). To enhance understanding of the relationships, we collected information in clinical trials (ClinicalTrials) (https://clinicaltrials.gov/) (37) and public literature MEDLINE (https://www.nlm.nih.gov) and PubTator (https://www.ncbi.nlm.nih.gov/CBBresearch/Lu/Demo/PubTator/) (38).

### Meta database construction: normalization of terminologies

To allow for interoperability and to bridge the research and the clinical settings, PreMedKB provided a wide variety of vocabularies in the meta databases. Standard names and synonymies were retrieved from different resources. Taking the construction of the drug meta database as an example, standard names (generic names) and synonymies (chemical names, trade names etc.) were firstly sourced from DrugBank (32) and Drugs@FDA. In order to collect a complete lexicon of drug synonymies, other names were then integrated from PubChem (33), STITCH (34) and UMLS (21). A standard name and its synonymies were assigned to the same drug ID in the drug meta database; therefore, our knowledgebase can accept different types of drug names as queries. The matching steps between standard names and synonymies were as follows: (i) general string processing of drug names and synonymies, such as stemming and turning into lowercases; (ii) setting up a list of stop words in both general and professional common words (e.g. cream, capsule, and recombinant); (iii) string matching and (iv) manual correction.

### Domain knowledgebase integration: extraction of semantic relationships

Due to the enormous number of domain knowledgebases in the biomedical research field, we chose several well-established databases, downloaded and parsed them into backend MySQL database separately, which makes it flexible to add, update and modify the contents in PreMedKB. Semantic relationships were extracted from different domain knowledgebases. A semantic relationship is defined as any relationship between two or more words based on the meaning of the words. In the simplest semantic relationship, it is composed of two words, a connection between the two words, and the word describing the type of the relationship and its direction. Many semantic relationships form a semantic network, and the nodes in the semantic networks are the words, whereas the edges are the connections (relationships). As the meta databases were constructed with a complete lexicon across the four components (diseases, genes, variants and drugs), nodes in the domain knowledgebases can be matched to meta database ID using the lexical matching method. Existing duplicated semantic relationships were removed, and a higher confidence rating was set for the node accordingly.

### Confidence ratings of semantic relationships

Confidence ratings of semantic relationships are essential to select the most related and important information in the knowledgebase. The ratings were calculated based on (i) the confidence of data sources and ratings in the original databases; (ii) the number of occurrence in diverse data sources; (iii) the number of relationships; (iv) the number and impact of supported clinical trials and publications and (v) manual revision. The range of confidence ratings was set between 1.0 (low confidence) and 5.0 (high confidence).

### Search builder design

#### Search portal

PreMedKB allows users to search by disease name, gene symbol (gene name), variant (variant loci, SNP ID, amino acid changes), drug name or combinations of these categories. PreMedKB offers fuzzy keyword searching capabilities, facilitating searches by returning the closest possible matching records.

#### Search strategy

In PreMedKB, the query term is firstly searched against the meta database to find its ‘standard name’. After going through the string processing, the best five matches of the query term are used to search for their semantic relationships. The search builder offers an open design which allows users to specify one or more keywords to find related nodes in PreMedKB. An explicit query can be easily built using multiple Boolean operators (and, or, not) with up to four terms. In the current version of PreMedKB, users are allowed to manually change the positions of brackets in the query-builder editor area to adjust the priority of the search terms.

#### Traverse level

Set the number of intermediate nodes existing between the hub and any of the node in a semantic network as *n*, the traverse level *l* between them is described as *n* + 1. In PreMedKB, the two nodes are represented by the query node and any specific node that is directly or indirectly related to it, respectively. When traverse level 1 is set (default), it means that the output is those directly related nodes with user's query. In order to show strongly associated results, PreMedKB allows users to search for nodes with up to three traverse levels, and high-confident but indirect links between nodes can be shown in the network.

#### Result ranking

The best five matches of the searched term are ranked according to how well they match with the query, and the semantic relationships between queries and other nodes are ranked according to the rating confidences. By default, the top 20 relationships are shown on the semantic network.

### Filtering methods

As the searching results may consist of many nodes and even more relationships, PreMedKB offers users filtering methods to remove (or hide) those parts that are less interesting. These filters are divided into two groups: by nodes, and by relationship. Users can filter out nodes and relationships by the node name and by relationship type or the relationship name. When selecting or unselecting some of the filters, the semantic network will be changed accordingly, and a sub-network of the whole semantic network will be displayed.

### Website design and database backend

PreMedKB consists of a collection of interconnected components, including a data server, a core RESTful backend central server that provides access to all data, and a front-end web server and web-based user interface (UI). The architecture makes the most of the three-level modeling approach (Model–View–Controller, MVC), where the storage can be selected independently of the high-level data access and representation, which also facilitates to access and represent data.

The web technologies implemented in the frontend web server and web-based UI include vis.js (http://www.visjs.org), D3 (https://d3js.org/), Integrative Genomics Viewer (IGV) (http://software.broadinstitute.org/software/igv/) (39), Raphaël (http://dmitrybaranovskiy.github.io/raphael/), and React framework (https://reactjs.org/). The vis is used to handle large amounts of dynamic data and to enable manipulation of and interaction with the data in the semantic network. The Charts of mutations distribution along the sequence, integrated genomic datasets and interactive bodymap are based on the library of IGV, Raphaël and D3, separately.

The data access service in PreMedKB is modeled as a resource-oriented architecture, which is based on the REST architectural style. REST is used to build distributed loosely coupled web services to address the needs of availability scalability and high performance in PreMedKB. All data are stored and managed using MySQL.

### Identifying genomic risk factors of a pancreatic cancer patient

A 49-year-old male patient was diagnosed with advanced pancreatic cancer after being mistreated as diabetes for 7 years. Whole-genome sequencing of the DNA from his blood sample was performed. DNA from peripheral blood lymphocytes was extracted, genomic library was then constructed, and sequencing was performed on an Illumina HiSeq X10 platform as paired-end 150-bp reads. Sample was sequenced to a depth of coverage ∼30×.

Read alignment and variant calling were performed using the DNAseq pipeline (Sentieon, Inc.) with the reference human genome GRCh37. Variants were annotated with genes and functions using ANNOVAR (9). After data filtering, two pathogenic candidate variants were identified and used as input of PreMedKB. Region covering this mutation was then confirmed using Sanger sequencing.

### Searching appropriate medicines for cancer patients

Somatic mutation profiles across 10 000 cancer patients were downloaded from Zehir *et al.* (17). Variants, genes containing non-synonymous variations and cancer type of each patient were used as input of PreMedKB. Drugs and confidence ratings showing the strength of evidences were obtained from PreMedKB. Drugs for targeted therapy were selected for further statistics.

## RESULTS

### Statistic summary and overview

PreMedKB is built to provide a resource for integrating information on diseases, genes, variants, drugs, and the relationships between any two or more of these four components with an important goal of facilitating the interpretation of the clinical meanings of a patient's genetic variants.

**Figure 2.**
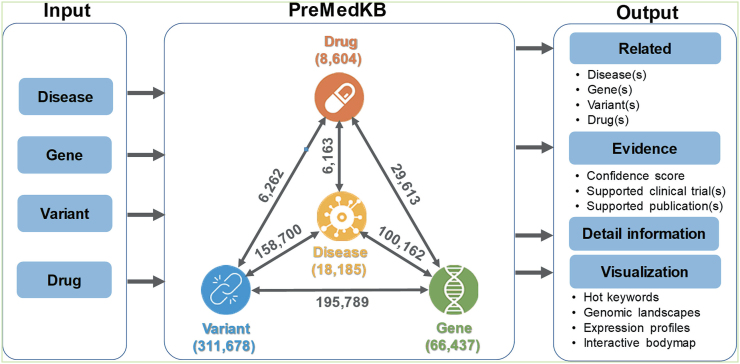
The schema of PreMedKB and its main features. PreMedKB provides a resource for integrating information on diseases, genes, variants, drugs, and the relationships between any two or more of these four components. PreMedKB allows users to search by disease(s), gene(s), variant(s), drug(s) or combinations of these categories. A comprehensive overview of the relationships between four components with evidences can be obtained with viewing facilities to help understanding the relationships.

The knowledgebase combines data from multiple sources and illustrates the confidence of the relationship through a user-friendly interface. PreMedKB currently consists of 18 185 diseases, 66 437 genes, 311 678 variants and 8604 drugs. Of these, a total of 496 689 relationships between 11 896 diseases, 17 698 genes, 195 688 variants and 6120 drugs have been curated. The overview and main features of PreMedKB are shown in Figure 2. A detailed summary is available on the ‘Statistics’ page at the PreMedKB website.

### User interface

**Figure 3.**
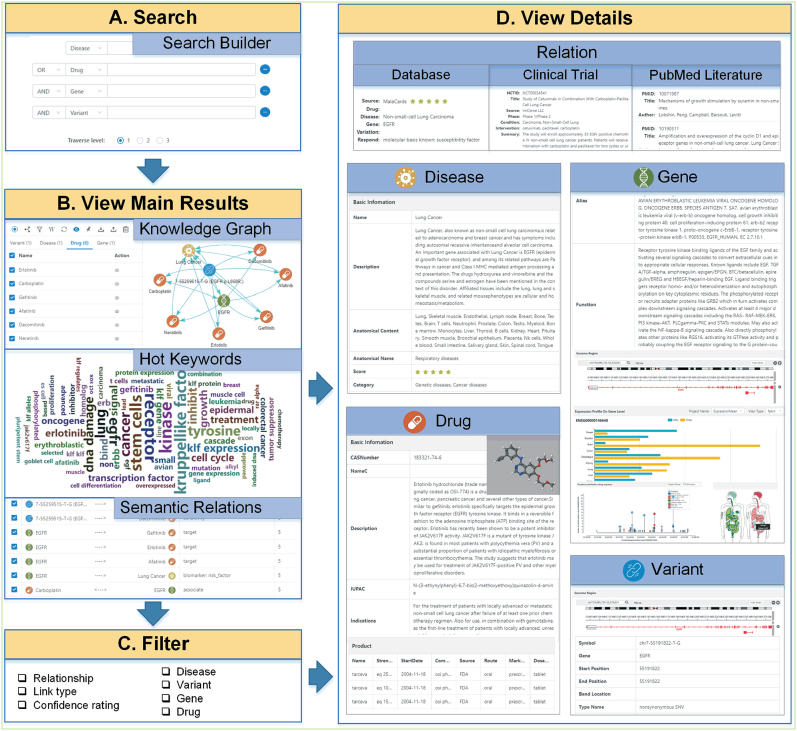
PreMedKB user interface. (**A**) Search builder of PreMedKB. PreMedKB provides search builder to accomplish complex search requirements. (**B**) As a result, a knowledge graph displaying diseases, genes, variants, and drugs that are related to the input query can be obtained. A word cloud showing hot keywords and a relationship table of semantic relations are also provided. (**C**) The interface enables sorting and filtering the resulting associations by rating, type of relations, specific diseases/genes/variants/drugs. (**D**) Each node and edge can be clicked to view detailed information. For edges, the source databases, related clinical trials and literature supporting the relationships are shown, and for nodes, the metadata can be displayed. In addition to showing the metadata in the ordinary way, we apply dynamic charts and interactive bodymap to visualize the mutation landscapes, expression profiles, gene locations and the 3D structure of the drug molecule.

PreMedKB provides a user-friendly web interface that enables users to search and retrieve all relationships among the four components in the database. PreMedKB allows users to search by using different types of identifiers and Boolean operators (Figure 3A). It provides a semantic network (knowledge graph) consisting of nodes and edges, displaying diseases, genes, variants and drugs that are related to the input query (Figure 3B). To help interpretation, users can view, select, move or delete (hide) nodes to modify the layout of the semantic network. Furthermore, the interface enables sorting and filtering the resulting network by rating, types of relationships, relationship names, and specific diseases/genes/variants/drugs (Figure 3C). Each node and edge can be clicked to view detailed information (Figure 3D). For edges, source database, related clinical trials and literature supporting the relationships are shown; and for nodes, metadata can be displayed.

In addition to showing the metadata in the ordinary way, we apply dynamic charts and interactive bodymap to visualize the mutation landscapes, expression profiles, gene locations, and the 3D structure of the drug molecule. A word cloud (hot keywords) showing text frequency and a relationship table of the semantic network are provided in PreMedKB. Results can be downloaded in txt/json/png/jpg format. Finally, a detailed tutorial on how to use PreMedKB is available on the ‘FAQ’ page at the PreMedKB website.

### Advantages of PreMedKB

We compared PreMedKB with CIViC (10), DrugBank (32), HGMD (11), My Cancer Genome, OncoKB (16), PanDrugs (40) and PharmGKB (13) (Table 2), and the advantages of PreMedKB are summarized below.

**Table 2.** Comparison between PreMedKB and other precision medicine databases

Databases	Knowledge field	Term Normalization	Search methods	Structured data	Knowledge presentation	Docking with NGS pipelines	Other information	Programmatic use	Data download
**CIViC**	Target therapy in cancer	No	Controlled words and free text	No	Tables and texts	Need format change	Meta data on gene and variant	Via API	Yes
**DrugBank**	Drug data and related target information	Yes	Free text	No	Tables and texts	Without specific variant sites	Literature, clinical trials, meta data on gene and drug	Via API	Yes
**HGMD**	Variants for human inherited disease	Yes	Database retrieval	No	Tables and texts	Direct	Literature	Via MySQL	License required
**My Cancer Genome**	Targeted therapies, immune therapies and other in cancer	No	Controlled words	No	Tables and texts	Need format change	Literature, clinical trials	No	No
**OncoKB**	Target therapy in cancer	Genes are normalized	Free text	Yes	Tables and dynamic graphs	Need format change	Literature, clinical sequencing cohort	Via API	Yes
**PanDrugs**	Target therapy, pharmacogenomics and drug repurposing in cancer	No	Controlled words	Yes	Tables and texts	Without specific variant sites	Cross links to NCBI.Gene, PubChem, PubMed and ClinicalTrials.gov	Via API	Yes
**PharmGKB**	Pharmacogenomics	No	Controlled words and free text	No	Tables and texts	Need format change	Literature	No	License required
**PreMedKB**	Target therapies, immunotherapies, chemo therapies, pharmacogenomics and pathogenic sites in cancer and other diseases	Yes	Free text and search builder	Yes	Tables, text and dynamic network / graphs	Direct	Literature, clinical trials, expression profiles and genomic landscapes	No	No

#### Comprehensive data coverage

By integrating over 20 public databases and employing semantic network techniques, PreMedKB contains rich biomedical knowledge of 496,689 relationships among disease, genes, variants and drugs. Specifically, it integrates databases of human variants in cancer and other genetic diseases, targeted and traditional treatments, drug responses and so on. In addition, transcriptomic landscapes, mutation profiles, clinical trials and PubMed references displayed in dynamic graphs can act as additional information to provide supporting evidence and help understand related knowledge for precision medicine.

#### Data organization

Knowledge in plain text is manually or computationally broken into key words that are then organized into semantic relationships. Furthermore, terminologies in diseases, genes, variants, drugs, semantic relationships and other key words were normalized, allowing for data from diverse sources be expressed in a unified manner.

#### User-friendly search methods

PreMedKB accepts different types of names as search queries. Advanced search allows the user to build specific searching strategies to suit for different study designs. And search results can be ordered according to how well they match the query.

#### Powerful data visualization

Semantic network and plug-ins are applied in order to demonstrate the complex searching results dynamically. In addition to showing information in tables or graphs, relationships are displayed in the form of networks, which makes it possible to discover indirect links among diseases, genes, variants and drugs, and to visually identify central hubs. Users can order the hubs and links and filter out those that are less interesting.

#### Progressive filtering mode

PreMedKB offers users the progressive filtering mode to add or remove nodes and relationships to the semantic network. By applying this method, users can quickly and precisely find out what they care about the most.

#### Straightforward data integration

Based on the normalized data, PreMedKB can flexibly dock with other third-party systems. Importantly, PreMedKB allows users to extract knowledge they are interested in by conveniently importing relationships in json format or exporting resulted relationships in txt/json/png/jpg format.

## EXAMPLES OF USE

Here we provide three examples of use of PreMedKB: i) learning molecular traits of lung cancer; ii) molecular diagnosis and identifying genomic risk factors; and iii) selecting appropriate medicines for a cancer patient. Each of them represents an application direction: inference of the disease pathogenicity at the molecular level, disease diagnosis, and guidance on drug recommendation.

### Learning molecular traits of diseases

**Figure 4.**
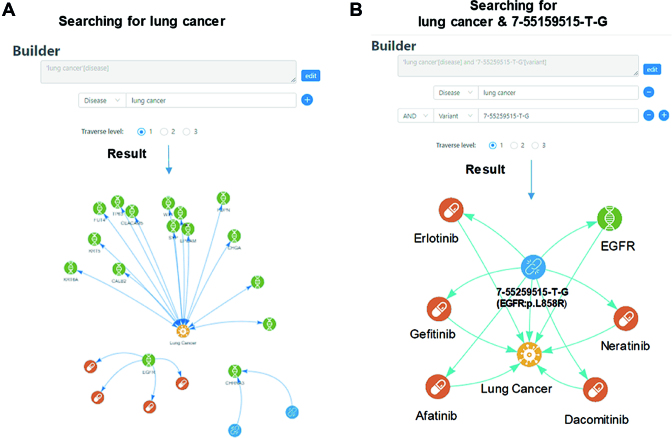
Examples of learning molecular traits of diseases by using single or combined searching query. (**A**) As single query search, ‘lung cancer’ is used as the search query to search for all directly linked nodes. By default, only the top 20 relationships are displayed. (**B**) As complex search, two queries ‘7-55259515-T-G’[variant] AND ‘lung cancer’[disease] is used. In the result, five drugs and their relationships with the two nodes in the search builder are shown in the semantic network.

A search builder is provided in PreMedKB so that it can be applied under different circumstances. Examples of searching for a single query and searching for more than one query are shown in Figure 4. In the example of searching for a single query, ‘lung cancer’ is used as the search query to search for all directly linked (traverse level = 1) nodes. After removing nodes that are not precisely related to ‘lung cancer’, 90 genes, 91 variants, 67 drugs as well as their relationships are shown in the semantic network (Figure 4A). These are lung cancer associated targets, pathogenicity, targeted therapies etc. By default, only the top 20 relationships are displayed.

In the example of searching for more than one query, users can search for nodes satisfying multiple conditions simultaneously. For example, drugs associated with EGFR:p.L858R in lung cancer are searched with the builder ‘7-55259515-T-G’[variant] AND ‘lung cancer’[disease]. In the results, five drugs and their relationships with the two nodes in the search builder are shown in the semantic network (Figure 4B). All these five drugs are targeted therapies for lung cancer patients carrying EGFR:p.L858R mutation. Among them, gefitinib (Iressa) and erlotinib (Tarceva) are first-generation tyrosine kinase inhibitors, whereas afatinib (Gilotrif), dacomitinib, and neratinib are second-generation inhibitors. All of them have been approved by the US FDA, represented by the high scores in each relationship.

Above all, the search builder allows users to check for general and specific results of their query terms. It is designed to help users deal with flexible search requirements.

### Molecular diagnosis and identifying genomic risk factors

PreMedKB can be used in analyzing clinical next-generation sequencing cases. A crucial step in such analyses is gene-phenotype interpretation, which is performed subsequent to initial sequence alignment, variant calling, annotation, and filtering (41). However, the most challenging aspect of NGS test is effective and comprehensive interpretation. PreMedKB provides an effective and user-friendly system for the interpretation of a patient's genetic variants.

**Figure 5.**
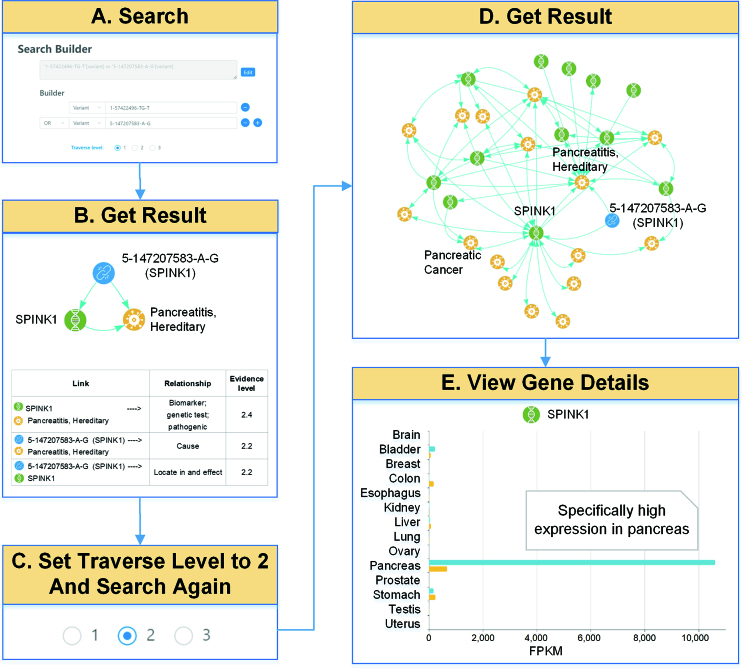
An example using PreMedKB to identify genomic risk factors in a pancreatic cancer patient. (**A**) After data analysis, two pathogenic candidate variants are identified and used as input of PreMedKB. (**B**) Variation of *SPINK1* (5-157828020-A-G) is identified as a cause of hereditary pancreatitis. (**C**) The traverse level is selected to be 2, showing an extensive search using the initial result as input. (**D**) A more comprehensive network is obtained. After filtering, *SPINK1* is shown to have a direct connection with pancreatic cancer and hereditary pancreatitis. (**E**) Detail information helps us understand the functions and expression profile of *SPINK1* gene.

Figure 5 shows an example how PreMedKB helped identify risk factors of a pancreatic cancer patient. This 49-year-old male patient was diagnosed with advanced pancreatic cancer after being mistreated as diabetes for seven years. Whole-genome sequencing of blood samples of the patient was performed. After data analysis, two pathogenic candidate variants were identified and used as input of PreMedKB (Figure 5A). Point mutation of the splicing site of *SPINK1* (5-157828020-A-G) was identified as a cause of hereditary pancreatitis (Figure 5B). The traverse level was selected to be 2, showing an extensive search using the initial result as input (Figure 5C). A more comprehensive network was obtained. *SPINK* was shown to have a direct connection with pancreatic cancer (Figure 5D).

As shown in gene detailed information page in PreMedKB (Figure 5E), *SPINK1* gene is specifically highly expressed in normal pancreas. The function of *SPINK1* is ‘…a trypsin inhibitor, its physiological function is to prevent the trypsin-catalyzed premature activation of zymogens within the pancreas. … is synthesized by several tumors and cell lines. … Elevated serum and urine levels occur particularly with mucinous ovarian cancer and may occur in nonmalignant diseases, e.g., pancreatitis…’, indicating that SPINK1 plays an important role in cancer.

We identified the point mutation of the splicing site in *SPINK1* as the possible genomic risk factor of the pancreatic cancer patient. The rationale is as follows:

- The variation may cause loss of function to SPINK1 gene. The variant (5-157828020-A-G) is predicted to lead to either an abnormal message that is subject to nonsense-mediated mRNA decay, or to an abnormal protein product if the message is used for protein translation.
- The variation is recognized as a cause of hereditary pancreatitis. The 5-157828020-A-G pathogenic variant in the SPINK1 gene has been reported to be association with hereditary pancreatitis (11). It rarely occurs in normal people (0.08% in 1000 Genomes Project and 0.04% in ExAC), but common in pancreatitis patients (57.43%) (42).
- Germline mutation of SPINK1 is recognized in cases of hereditary chronic pancreatitis and incidence of pancreatic cancer (43–45).
- As the patient was mistreated as diabetes for 7 years. In fact, the patient's pancreatic function of insulin release was compromised due to hereditary pancreatitis, showing symptoms similar to those of diabetes.
- Technically, the variation is validated using independent Sanger sequencing technology.

Thus, although limited publications show relationships between the variant and pancreatic cancer, PreMedKB greatly helped us recognize the variation in *SPINK1* as the genomic risk factor of this 49-year-old pancreatic cancer patient.

### Selecting appropriate medicines for a cancer patient

PreMedKB can be used to provide precision treatment options to help clinicians make a clinical decision based on an individual's genomic data. The performance of the PreMedKB system has been compared favorably with that of OncoKB used for interpreting the MSK-IMPACT data set of genetic profiles of about 10 000 cancer patients (16,17).

After variant annotation and filtering, PreMedKB reported that a total of 40% of patients were found to carry at least one treatment-associated genetic alteration (only focusing on target therapies in accordance with the MSK-IMPACT study). 28% of them carry clinically actionable variants where targeted therapy has already been approved in the US or China, or has become standard of care, whereas 12% patients carry variants with less strong evidences of care. In the original publication of the MSK-IMPACT study with OncoKB as the interpretation tool, 36% of patients were found to carry at least one treatment-associated genetic alteration (level 1, level 2 and level 3) and 18% of patients were found to carry variants for which targeted therapy has been approved by the US FDA (level 1 and level 2).

Unfortunately, it is difficult to perform a head-to-head comparison of the clinical interpretation of the patients’ genomic profiles between PreMedKB and OncoKB. One important reason is that the clinical interpretation was not provided by OncoKB at an individual patient level (17).

## CONCLUSION AND FUTURE PERSPECTIVES

In summary, PreMedKB is an efficient and user-friendly tool to assist researchers, clinicians or patients in interpreting a patient's genetic profile in terms of discovering potential pathogenic variants or investigating the molecular basis of specific diseases, recommending therapeutic regimens, designing panels for genetic testing kits, matching patients for clinical trials, and gaining insight into any of the four elements that are of interests to the users. PreMedKB integrates verified and normalized knowledge from well-established databases from different fields, employs technologies in the semantic network, and offers flexible search strategies with search portals in disease, gene, variant and drug to obtain verified, normalized and structured knowledge at once efficiently. In addition, PreMedKB can dock with other platforms to solve complex scientific problems that require broader knowledge. Customized applications can be developed by re-organizing knowledge from PreMedKB.

PreMedKB will be continuously updated. In addition to integrating more domain knowledgebases and meta databases required in clinical data interpretation, we will focus on taking advantages of these lines of information to design more comprehensive search approaches. For example, by upgrading the APIs in searching symptoms related a disease, we will be able to look for potential risk factors underlying a certain symptom and recommend potential therapies. Moreover, we will try to design APIs or applications to dock with NGS pipelines and other platforms, and to better update database information in a timely fashion. We would like PreMedKB to be a better hierarchical data resource so that more precise knowledge will be offered.

## Notes

**Disclaimers**: PreMedKB should be considered as a research tool only; use of the information from PreMedKB for medical purposes is discouraged and the user should seek a physician's advice on the suitability of the information in PreMedKB.
